# Progranulin Adsorbs to Polypropylene Tubes and Disrupts Functional Assays: Implications for Research, Biomarker Studies, and Therapeutics

**DOI:** 10.3389/fnins.2020.602235

**Published:** 2020-12-14

**Authors:** Sushmitha Gururaj, Paul J. Sampognaro, Andrea R. Argouarch, Aimee W. Kao

**Affiliations:** Memory and Aging Center, Department of Neurology, University of California, San Francisco, San Francisco, CA, United States

**Keywords:** progranulin, adsorption, neurodegeneration, polypropylene, cathepsins

## Abstract

Progranulin (PGRN) is a tightly regulated, secreted glycoprotein involved in a wide range of biological processes that is of tremendous interest to the scientific community due to its involvement in neoplastic, neurodevelopmental, and neurodegenerative diseases. In particular, progranulin haploinsufficiency leads to frontotemporal dementia. While performing experiments with a HIS-tagged recombinant human (rh) PGRN protein, we observed a measurable depletion of protein from solution due to its adsorption onto polypropylene (PPE) microcentrifuge tubes. In this study, we have quantified the extent of rhPGRN adsorption to PPE tubes while varying experimental conditions, including incubation time and temperature. We found that ∼25–35% of rhPGRN becomes adsorbed to the surface of PPE tubes even after a short incubation period. We then directly showed the deleterious impact of PGRN adsorption in functional assays and have recommended alternative labware to minimize these effects. Although the risk of adsorption of some purified proteins and peptides to polymer plastics has been characterized previously, this is the first report of rhPGRN adsorption. Moreover, since PGRN is currently being studied and utilized in both basic science laboratories to perform *in vitro* studies and translational laboratories to survey PGRN as a quantitative dementia biomarker and potential replacement therapy, the reported observations here are broadly impactful and will likely significantly affect the design and interpretation of future experiments centered on progranulin biology.

## Introduction

Progranulin (PGRN) is an evolutionarily conserved, cysteine-rich, secreted glycoprotein with a diverse set of functions. The pleiotropic nature of PGRN is evidenced by a growing body of work that details its participation in important biological processes such as vascular protection (Kanazawa et al., 2015), neuronal connectivity and survival (Gass et al., 2012; Ward et al., 2014), wound healing (Zhu et al., 2002), inflammation (Jian et al., 2013; Tanaka et al., 2013; Minami et al., 2014; Xu et al., 2016), and immunity (Matsubara et al., 2012; Jian et al., 2013). In recent years, it has become apparent that cells maintain tight regulation of PGRN levels. Excessive PGRN production is associated with cancers that grow aggressively and metastasize early (He and Bateman, 1999; Yang et al., 2015; Serrero et al., 2016), while PGRN haploinsufficiency and homozygous loss of function states lead to adult-onset frontotemporal dementia (FTD) (Baker et al., 2006; Cruts et al., 2006) and childhood-onset neuronal ceroid lipofuscinosis (NCL) (Smith et al., 2012), respectively. Intriguingly, PGRN gene (*Pgrn*) delivery has also demonstrated ameliorative effects in animal models of both Alzheimer’s (Van Kampen and Kay, 2017) and Parkinson’s disease (Van Kampen et al., 2014).

Based on these observations, circulating PGRN has garnered recent attention as a potential quantitative biomarker for neurodegeneration, particularly for FTD. *Pgrn* mutations causing FTD manifest in reduced plasma, serum, and cerebrospinal fluid (CSF) PGRN protein levels compared to non-mutation carriers (Ghidoni et al., 2008; Finch et al., 2009; Sleegers et al., 2009; Nicholson et al., 2014; Meeter et al., 2016; Wilke et al., 2017). Perhaps the strongest argument for the consideration of PGRN levels is that the clinical diagnosis of FTD lies in recent clinical trials testing PGRN replacement therapy, in which successful dose-dependent increases in plasma and CSF PGRN are already being reported (NCT03636204, NCT03987295). However, discrepancies exist within the current literature. For example, reduced serum PGRN do not seem to correlate tightly with reduced PGRN levels in the CSF of both Pgrn ± and Pgrn−/− FTD patients, suggesting either differences in peripheral PGRN regulation or potential sources of measurement error in PGRN quantification between these sample types (Wilke et al., 2016).

Our laboratory studies PGRN and its impact on lysosome biology and protein homeostasis (Salazar et al., 2015; Butler et al., 2019a,b,c). In several of our biochemical assays involving human plasma and CSF, we routinely use an ∼80kDa recombinant HIS-tagged human Progranulin (rhPGRN) protein as a control for the full-length human PGRN protein. While performing these experiments, we observed inconsistencies in our rhPGRN control measurements in enzyme-linked immunosorbent assays (ELISA) and mass spectrometry assays. Troubleshooting these inconsistencies led to the discovery of an important caveat of working with this recombinant protein: PGRN depletes from solution by adsorbing to the polypropylene surface of microcentrifuge tubes. Given the nearly universal presence of polypropylene and other plastic labware in experimental workflows, we performed a detailed characterization of rhPGRN adsorption to polypropylene tubes. This study quantifies the adsorption of PGRN to polypropylene tubes, demonstrates the functional effects of this adsorption, and recommends alternatives to polypropylene to counteract it. Given the significant evidence for PGRN’s tight *in vivo* regulatory control and in light of recent diagnostic and therapeutic efforts aimed at modulating PGRN levels in patients (particularly, those with neurodegenerative disease), we present these data in an effort to optimize future work in these areas.

## Materials and Methods

### Materials

Labware–1.5 mL polypropylene microcentrifuge tubes (E&K Scientific Products, No. 695054), 1.5 mL LoBind microcentrifuge tubes (Fisher Scientific Sci, No. E925000090), polypropylene pipet tips (United States Scientific, No. 1,111–1,700, 1–200 μL, low binding pipet tips (Sigma-Aldrich, No. CLS4151). Antibodies–anti-progranulin C-terminus (Thermo Fisher #40-3400, 1:1,000).

### BSA Coating of Polypropylene Tubes

BSA (Sigma, St. Louis, MO, United States) was dissolved in deionized water at a concentration of 100 mg/mL and sterile filtered. 100 μl of the BSA solution was incubated in the 1.5 mL polypropylene tubes for 24 h at room temperature. The solution was then aspirated, spun, and all remaining solution was aspirated.

### PGRN Adsorption Experiments

Recombinant human PGRN (R&D Systems Inc., No. 2420-PG-050) was prepared to the required concentration in Dulbecco’s Phosphate Buffered Solutions (DPBS) (Thermo Fisher Scientific, Waltham, MA, United States). Experiments were performed in 1.5 mL polypropylene tubes, Lobind tubes, or BSA-coated polypropylene tubes. Low binding pipet tips were used in all experiments except in the pipetting loss experiments, where polypropylene tips were used. 30 μl of 100 nM PGRN solution was made in each tube or in the control tube for the serial transfer and pipetting experiments and 30 μl of 250, 150, 100, 75, 50, or 25 nM was made in the dilution series experiments. 10′ incubations were performed on ice, unless specified as in the temperature and time experiments. To analyze PGRN in solution, either aliquots (5 μl) or the entire 30 μl volume were removed as indicated by the figure legends, and mixed with 4X lithium dodecyl sulfate (LDS) (Thermo Fisher Sci, No., NP0007) and 10X reducing agent (Thermo Fisher Sci, No. NP0009). These sample mixtures were boiled and visualized by Western blot. Adsorbed PGRN was analyzed by aspirating all the remaining solution from the tubes, adding 4X Laemmli buffer and 10X reducing agent, and boiling the tubes. Each tube was vortexed before and after boiling, and PGRN was then visualized by Western blot.

### *In vitro* Cleavage Assays

Recombinant human PGRN (R&D Systems Inc., No. 2420-PG-050) was prepared in DPBS (Thermo Fisher Scientific, Waltham, MA, United States). Experiments were performed in 1.5 mL polypropylene tubes and Lobind tubes. Low binding pipet tips were used in all experiments. 400 ng of rhPGRN was incubated with 250 nM of Cathepsin L (R&D Systems, No. 952-CY-010) in the presence of sodium acetate buffer (pH 4.5), 10 μM of dithiothreitol (DTT), and 10 μM of ethylenediaminetetraacetic acid (EDTA) for 2.5, 5, 10, and 15 min. These timed incubations were quenched by the addition of 4X LDS (Thermo Fisher Sci No., NP0007) and 10X reducing agent (Thermo Fisher Sci, No. NP0009), and placed on ice. These sample mixtures were then boiled and visualized by Western blot.

### Western Blots

Progranulin was visualized by reducing (sodium dodecyl sulfate–polyacrylamide gel electrophoresis) SDS-PAGE using 4–12% Bis-Tris gels (Thermo Fisher sci, No. NP0302BOX). Proteins were transferred to PVDF membrane (Bio-Rad, No. 1620177) and blocked with 5% milk (Thermo Fisher, No.NC9121673) or Odyssey buffer (Li-cor No. 927-50010) for 2 h at room temperature. Membranes were incubated in primary antibodies overnight at 4°C. Li-cor secondary antibodies were used at 1:5,000 dilution for 1 h at room temperature and blots were imaged using the Odyssey CLx imager.

### Analysis of Western Blots

**FIGURE 1:**
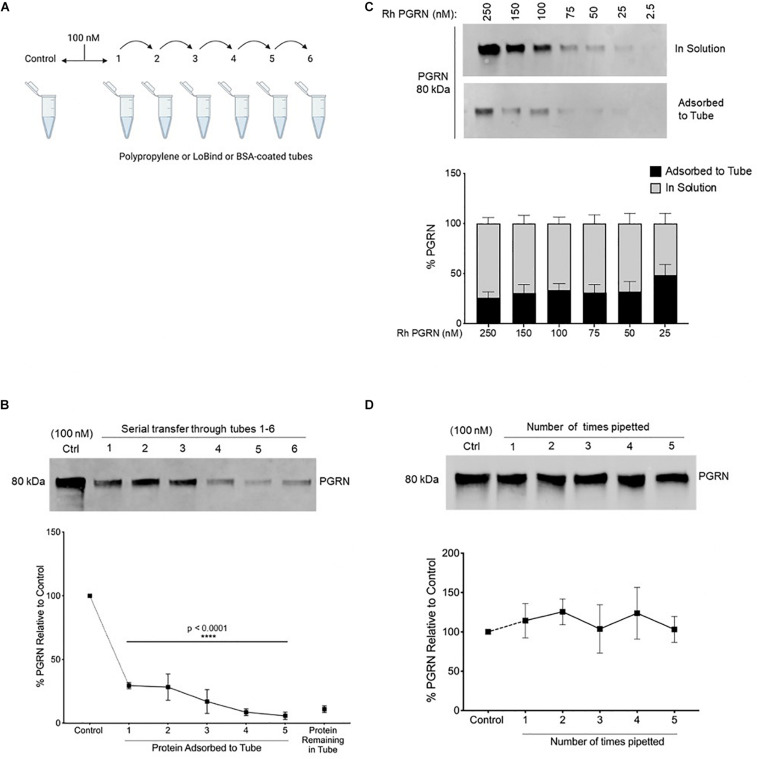
Recombinant Progranulin Protein Adsorbs to Polypropylene Tubes. **(A,B)** 100 nM rhPGRN was serially transferred through tubes 1–5, which were stripped for adsorbed protein after a 10-min incubation in each tube. Tube 6 contains the protein remaining in solution at the end of five serial transfers. The control is 100 nM PGRN that underwent no transfers. Low binding pipet tips were used to minimize pipetting loss. One-Way ANOVA with Holm-Sidak’s Multiple Comparisons test was performed (****p < 0.0001, 1–6 vs. Control, *n* = 3). **(C)** A dilution series of 250–25 nM PGRN was set up in polypropylene tubes. After a 10-min incubation on ice, the PGRN in solution as well as adsorbed to every tube was prepared for analysis. Low binding pipet tips were used to minimize pipetting loss. **(D)** 100 nM PGRN was prepared in tubes 1–6. Polypropylene tips were used to aspirate and dispense the full volume of solution 0–5 times, respectively, into the same tube. The control is 100 nM PGRN subjected to no pipetting. PGRN levels were measured by Western blot and membranes were immunoblotted with anti-PGRN antibody. All immunoblots are representative of three independent experiments.

In order to quantify the relative intensity of bands on Western Blots, the captured images from the Odyssey CLx were subject to analysis using ImageJ (Rasband, W.S., ImageJ, United States National Institutes of Health, Bethesda, MD, United States, 1997–2012)^1^. For Figures 1A,B, % PGRN values were obtained by normalizing to the control band signal. For all of the other figures, % PGRN detected either in solution or adsorbed to the tube for a particular condition was calculated as a fraction of the total signal: {[intensity of in solution or adsorbed to tube band/(intensity of in solution + adsorbed to tube) bands] × 100}.

### Statistics

All results shown are representative of three independent experiments. All statistics were performed using GraphPad Prism Version 6 (GraphPad Software, La Jolla, CA, United States). For comparisons with control, One-way ANOVA followed by Holm-Sidak’s multiple comparisons test was used. For comparisons in all other figures, the difference between protein levels in solution and adsorbed to the tube at a given condition were calculated by paired *t*-tests while differences between the conditions within the in solution and adsorbed groups were calculated by One-way ANOVA followed by Dunnett’s multiple comparisons test. In all graphs, error bars indicate the standard deviation of the data. *P* values for the concerned tests are as specified in figure legends.

## Results

### rhProgranulin Protein Adsorbs to Polypropylene Tubes

It is well established that most proteins and peptides readily bind to experimental surfaces owing to their amphipathic nature (Nakanishi et al., 2001). This very property is exploited beneficially in several bio-techniques, such as the ELISA immuno-assay where the coating of 96-well plastic plates relies on non-specific adsorption, and ion exchange chromatography whose central principle is the binding between charged proteins and resins. However, adsorption can exert undesirable impacts on quantitative protein studies and can lead to inaccurate working concentrations. We observed in our experiments that the incubation of rhPGRN protein in PPE microcentrifuge tubes depleted the protein amounts in solution over time. Therefore, we hypothesized that the rhPGRN was becoming adsorbed to polypropylene tubes.

To quantitatively assess rhPGRN adsorption to polypropylene tubes, 100 nM rhPGRN was incubated in tubes on ice for 10 min, then serially transferred and incubated for 10 min in a new tube; this procedure was subsequently repeated four more times. Each empty tube was stripped of adsorbed PGRN by boiling with reducing Laemmli buffer and run on SDS-PAGE gels, along with the entirety of the rhPGRN left in tube 6 (Figure 1A). Pipetting loss of protein was minimized in these experiments by using low binding pipette tips. Upon comparison with a control sample that underwent no transfers, ∼10–25% of PGRN was found to become adsorbed to a tube with each incubation such that after five transfers, only ∼15% of the protein remained in solution (*p* < 0.0001) (Figure 1B). We also measured this phenomenon via ELISA and found similar results (Supplementary Figure 1).

Next, we asked if the concentration of PGRN affects the extent of adsorption; for example, if concentrations lower than 100 nM would be entirely depleted from solution or if higher concentrations might be found in solution still due oversaturation of the adsorption surface. A concentration series (250–25 nM) was set up in polypropylene tubes for 10-min incubations and the adsorbed and in-solution PGRN were measured (Figure 1C). Within the tested concentration range, ∼25–35% of protein was consistently found to be adsorbed across all concentrations.

We further examined PGRN loss to tube adsorption as a result of repeated pipetting during routine aspiration and/or dispensing of the solution in experiments. To test this, we prepared 100 nM PGRN solution in polypropylene tubes as previously described but instead of serially transferring the solution into new tubes, we reapplied it to the same tube with a fresh 100 μL polypropylene pipet tip. Samples 1 through 5 were prepared by sequentially increasing the number of times the full volume was aspirated and dispensed with a single tip from one to five, respectively (Figure 1D). When compared to a control preparation that was not subjected to pipetting, we found that pipetted samples underwent no measurable adsorption loss. The pipetting and incubation experiments differ primarily in the time of exposure of the sample to the plastic. The exposure time of the pipette tip’s binding surface is only a few seconds whereas samples were incubated for 10 min in the tubes. Taken together with the previous results, in experimental practice it appears that polypropylene tubes, but not pipet tips, lead to measurable PGRN adsorption to labware under the conditions tested.

### Time and Temperature Impact Adsorption of Recombinant Progranulin to Polypropylene Tubes

**FIGURE 2:**
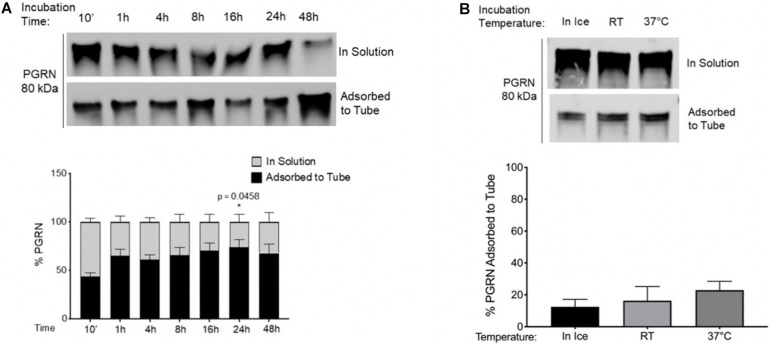
Time and Temperature Impact Adsorption of Recombinant Progranulin to Polypropylene Tubes. **(A)** 100 nM PGRN was incubated for 10′, 1, 4, 8, 16, 24, or 48 h in polypropylene tubes on ice. One-Way ANOVA with Dunnett’s Multiple Comparisons were performed (*p < 0.05, 1/4/8/16/24/48 h vs. 10′, *n* = 3). **(B)** 100 nM PGRN was incubated in polypropylene tubes for 10′ on ice, at room temperature (RT) or at 37°C (*n* = 3). At the end of the incubations, PGRN in solution as well as adsorbed to the tube was prepared for analysis. PGRN levels were measured by Western blot and membranes were immunoblotted with anti-PGRN antibody. All immunoblots are representative of three independent experiments.

Given the aforementioned findings, as well as the fact that experimental conditions such as incubation time and temperature are often important factors in study design, we examined if rhPGRN adsorption to polypropylene tubes is influenced by (1) duration of incubation and (2) temperature. We also briefly looked at frozen storage (Supplementary Figure 2). To study incubation time as a factor, adsorbed and in-solution PGRN amounts were measured at the previously tested 10-min incubation timepoint, but also after 1, 4, 8, 16, 24, and 48 h of incubation on ice. Although the majority of PGRN adsorption seemed to occur rapidly with ∼40% adsorbed at the end of 10 min, we observed a trend toward further protein absorption over time, including a statistically significant increase of ∼25–35% additional starting protein found adsorbed to the tube at 24 h (*p* = 0.0458) (Figure 2A). It must be noted that this 40% adsorption is higher than was reported in previous 10-min incubations (Figures 1B,C). Indeed, we observed inter-experimental variation across our entire study and found the amount of adsorption to have a range as broad as 10–40% between experiments.

PGRN adsorption to PPE may also be temperature dependent. Incubation at room temperature (RT) or at 37°C for 10 min trended toward increasing PGRN adsorption when compared to ice-incubation (Figure 2B). These results demonstrate that the considerable amount of basal PGRN adsorption observed under conventionally used experimental guidelines for recombinant proteins is further increased by varying conditions such as duration or temperature of incubations, necessitating additional caution among experimenters when designing protocols with PPE tubes.

### PGRN Adsorption Is Reduced in Low Binding and BSA-Coated Tubes

**FIGURE 3:**
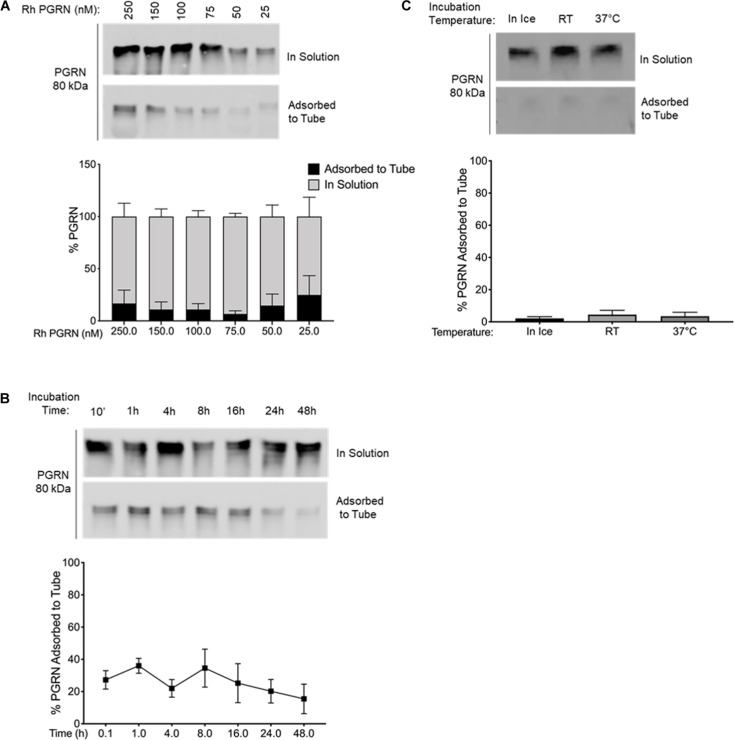
PGRN Adsorption is Prevented in BSA-Coated Tubes. **(A)** A dilution series of 250–25 nM PGRN was set up in BSA-coated polypropylene tubes for 10-min incubations on ice (*n* = 3). **(B)** 100 nM PGRN was incubated for 10′, 1, 4, 8, 16, 24, or 48 h in BSA-coated tubes on ice (*n* = 3). **(C)** 100 nM PGRN was incubated in BSA-coated tubes for 10′ on ice, at room temperature (RT) or at 37°C (*n* = 3). At the end of the incubations, PGRN in solution as well as adsorbed to the tube was prepared for analysis. PGRN levels were measured by Western blot and membranes were immunoblotted with anti-PGRN antibody. All immunoblots are representative of three independent experiments.

**FIGURE 4:**
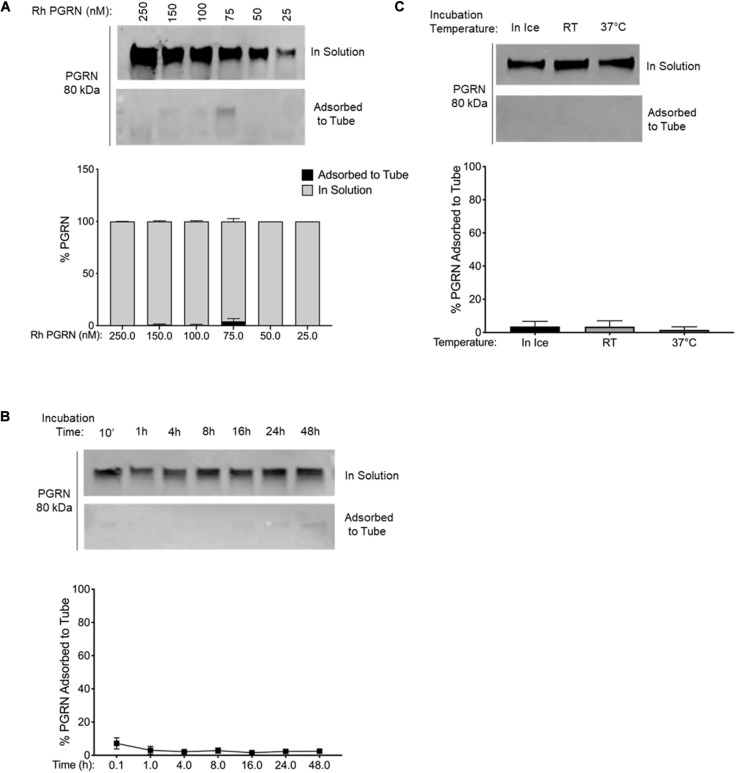
PGRN Adsorption is Reduced in LoBind Tubes. **(A)** A dilution series of 250–25 nM PGRN was set up in LoBind tubes for 10-min incubations on ice (*n* = 3). **(B)** 100 nM PGRN was incubated for 10′, 1, 4, 8, 16, 24 or 48 h in LoBind tubes on ice (*n* = 3). **(C)** 100 nM PGRN was incubated in LoBind tubes for 10′ on ice, at room temperature (RT) or at 37°C (*n* = 3). At the end of the incubations, PGRN in solution as well as adsorbed to the tube was prepared for analysis. PGRN levels were measured by Western blot and membranes were immunoblotted with anti-PGRN antibody. All immunoblots are representative of three independent experiments.

We tested two alternatives to polypropylene tubes: (1) BSA-coated tubes prepared by incubating 100 mg/ml BSA in polypropylene tubes for 24 h at room temperature (Figure 3) and (2) commercially available low-binding “LoBind” tubes (Figure 4). As in a previous set of experiments, rhPGRN concentrations from 25 to 250 nM were incubated in LoBind or BSA-coated tubes for 10 min on ice. Overall, both LoBind and BSA-coated tubes performed better than polypropylene tubes, with BSA-coated tubes resulting in nearly complete retention of PGRN in solution across the concentration range (Figures 3A, 4A). LoBind tubes, on the other hand, resulted in lesser PGRN adsorption loss than polypropylene tubes (∼10–15 vs. ∼25–35%), but did not completely prevent it.

Additionally, we examined if the two alternative tubes would fare better under conditions that exacerbated rhPGRN adsorption loss in polypropylene tubes, namely longer incubation times and higher incubation temperatures. We found that PGRN adsorption did not increase in a time- or temperature-dependent manner in LoBind (Figures 3B,C) or BSA-coated tubes (Figures 4B,C). However, a measurable amount of PGRN adsorption did occur in LoBind tubes at all the measured timepoints, consistent with previous results that it was but a slight improvement to polypropylene tubes. BSA coating, in contrast, prevented adsorption entirely under all tested conditions. Taken together, these results indicate that under the test conditions, BSA-coated tubes appear to be the most reliable at maintaining working concentrations of rhPGRN in solution, followed by LoBind tubes, and polypropylene tubes.

### PGRN Adsorption to PPE Tubes Disrupts a Functional Assay

**FIGURE 5:**
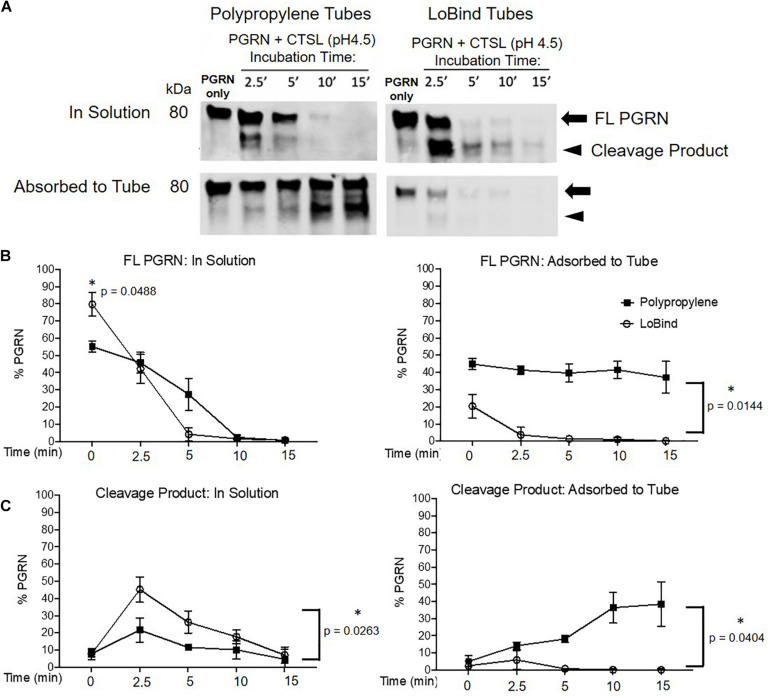
Protease Kinetics of Cathepsin L mediated PGRN Cleavage are Disrupted in Polypropylene Tubes. **(A)** 400 ng PGRN was incubated with 250 nM of Cathepsin L for 2.5, 5, 10 and 15 min in either polypropylene or LoBind tubes. **(B,C)** Quantification of the relative percentage of full-length PGRN and its approximately 75 kDa cleavage product after incubation with Cathepsin L for the aforementioned time points. Paired *t*-tests were performed (^∗^*p* < 0.05 in solution vs. adsorbed to tube for every condition, *n* = 3). All immunoblots are representative of three independent experiments.

Following the above described characterization, it was critical for us to determine if PGRN adsorption interferes with the results of a functional assay. We routinely study the dynamics of PGRN processing into granulins using *in vitro* protease cleavage assays with rhPGRN and commercially available recombinant human lysosomal proteases. Based on previous work identifying the endo-lysosomal enzyme Cathepsin L (CTSL) as a PGRN protease (Lee et al., 2017), we compared the results from dynamic rhPGRN cleavage assays between polypropylene and Lobind tubes (Figure 5). Because BSA is a known CTSL substrate, BSA-coated tubes were not included in this experiment. Here, rhPGRN was incubated with recombinant CTSL at pH 4.5 for 2.5, 5, 10, or 15 min at 37°C. At the end of each timepoint, the full reaction volume was transferred to a new tube and the protein in solution as well as adsorbed to the incubation tube were prepared for western blot analysis as previously described. In this functional assay, the observed PGRN cleavage product (approximately 75 kDa in size) was noticeably absent from solution when the reaction was performed within polypropylene tubes, particularly at the 2.5-min timepoint. Taken together, these results demonstrate that *in vitro* cleavage assay kinetics are also subject to artifactual changes depending on the amount of protein adsorbed to the tube.

## Discussion

This study demonstrates that the adsorption of rhPGRN to polypropylene tubes has the potential to create artifacts in working concentrations and hence impact the results of quantitative studies. This adsorption to polypropylene increases proportionally with increased duration of incubation and temperature. We show that using commercially available LoBind tubes and BSA coated polypropylene tubes can decrease or prevent adsorption, respectively.

Historically, common strategies to combat adsorption include adding detergents and increasing salt concentrations (Smith et al., 1978), coating surfaces with bovine serum albumin (BSA) (Felgner and Wilson, 1976) or polyethylene glycol (PEG) (Kramer et al., 1976), using siliconizing agents (Suelter and DeLuca, 1983), and choosing optimal labware (Goebel-Stengel et al., 2011). More recently, manufacturers have developed novel plastic polymers with lower protein retention properties than polypropylene for use in commercially available low binding “LoBind” tubes and pipette tips. Our comparative assessment of BSA-coated and LoBind tubes reveals BSA coating of polypropylene tubes to be an effective and inexpensive step toward preventing PGRN adsorption. The ability of BSA to block PGRN adsorption may be the result of a few factors. First, the polypropylene binding surface may achieve occupancy saturation by BSA and second, BSA may interact directly with the rhPGRN so as to facilitate PGRN protein remaining in solution. Protein adsorption theory suggests that in multi-component solutions, the former is dominant; a mass transfer phenomenon known as the Vroman Effect dictates that in a collection of proteins capable of electrostatically interacting with polypropylene, the most concentrated protein of the lowest molecular weight would arrive at the surface first, followed by the larger, less abundant ones (Fang et al., 2005). This likely underlies why protein adsorption has been described predominantly in single-component preparations of purified proteins and peptides. However, BSA contamination can be suboptimal and interfere in certain experimental designs, such as the *in vitro* cleavage assays described here. The presence of BSA (a known CTSL substrate) (Völkel et al., 1996) or any other contaminant in the reaction would likely disrupt interactions between the intended binding partners. Of note, our study has not exhausted the available coating alternatives such as glycine, poly-glycine or poly-alanine. Until future studies are performed testing such coating options, we recommend LoBind tubes for studies that preclude introduction of BSA into carefully optimized assay reactions such as ours.

However, the question of whether adsorption exclusively plagues purified protein preparations is worth asking. Complex human body fluids such as blood, plasma, and CSF are the most used sources for the measurement of endogenous PGRN by ELISA immuno-assays. As previously mentioned, inherent to the affordability of currently available plate-based assays is the use of polymer plastic 96-well plates. ELISAs of plasma and serum are likely buffered by albumin, immunoglobulins and other high concentration carrier proteins that effectively coat PPE tubes to prevent lower concentration protein binding to the surface. Indeed, the major human plasma proteins (albumin, γ-globulin, fibrinogen) are not only significantly more concentrated than PGRN by orders of magnitude, but also smaller by molecular weight, ensuring in theory that they would preferentially bind to the surface as per the Vroman effect (Anderson and Anderson, 2002). Additionally, sandwich ELISA assays such as those presently in the market for human PGRN consist of plates whose wells are coated with the capture antibody at the time of use. In combination, we believe that published reports of endogenous PGRN in human plasma and serum samples may suffer from adsorption-related errors to a negligible degree. However, it is entirely possible that protein adsorption accounts for some fraction of the reduced CSF PGRN levels found in recent studies (Wilke et al., 2016), given the decreased abundance of carrier proteins relative to blood preparations (Hühmer et al., 2006). In support, some instances of protein adsorption from complex biological samples onto tubes have been observed. For example, Aβ amyloid from mammalian and *C. elegans* extracts undergo adsorption to multi-well plates and Eppendorf tubes (Murray et al., 2013), and in ventricular CSF, 370 proteins including but not limited to the most abundant proteins were reported as adsorbed to a polycarbonate surface used for microdialysis (Undin et al., 2015). Considering that circulating PGRN is increasingly referenced to as a potential biomarker for FTD and is being tested as a potential replacement therapy in clinical trials, it is imperative that a characterization study similar to ours be done in human plasma, serum, and CSF.

So, what can be done? Until such a comparison study is conducted with endogenous PGRN, it is our recommendation that rigorously consistent experimental practices minimizing adsorption should be used for all *in vitro* PGRN research. Experimental steps and conditions that are routinely part of PGRN studies such as the transfer of samples between tubes, the duration of incubation of samples in tubes, and the incubation temperature, all play an important role in the maintenance of accurate working concentrations in solution. In parallel, we recognize the limitations of our recommendation, as demonstrated by our own limitations with using BSA-coated tubes in *in vitro* assays. Our group is one of many routinely using such assays to understand the proteolytic processing of PGRN into granulins, wherein reaction conditions are established with utmost care to facilitate interactions between rhPGRN and rh proteolytic enzymes. In such instances, we recommend the consistent use of LoBind tubes, which we have adopted in all of our studies.

It must be noted that while our study has characterized adsorption of one particular rhPGRN protein (R&D), several other recombinant PGRN proteins are commercially available. Future studies examining whether polymer binding is a ubiquitous property across all rhPGRN proteins and peptides may shed light on structure, and in turn, function and biology. As a start, certain unique properties of predicted PGRN structures lend toward an interesting hypothesis—the unusually high numbers of disulfide bridges may contribute to an inflexible exterior of hydrophobic residues, a known characteristic of “sticky” proteins. The predicted structure of PGRN may be theorized as sticky throughout given the distribution of disulfide bridges, however, future testing of truncated protein including multi-granulin peptides and individual granulin peptides would help confirm or deny this hypothesis.

In this work, we have observed that ∼25–40% of rhPGRN becomes adsorbed to the surface of polypropylene tubes when used for routine experimental use. This phenomenon may lead to unreliable quantification of rhPGRN and may create deleterious effects with PGRN functional assays. Our study deems it prudent for PGRN researchers, both at the bench and in the clinic, to pay close attention to experimental design, choice of optimal labware, and to prepare PGRN-containing samples in a cautious and consistent manner.

## Data Availability Statement

The original contributions presented in the study are included in the article/Supplementary Material, further inquiries can be directed to the corresponding author/s.

## Author Contributions

SG and PS designed all experiments, analyzed all the data, and contributed equally to this work. SG and AK conceived the project. PS performed the PGRN and CTSL *in vitro* experiments and western blots. AA assisted in performing several western blots. SG performed all other experiments. SG, PS, and AK wrote the manuscript. All authors have reviewed and approved this manuscript.

## Conflict of Interest

The authors declare that the research was conducted in the absence of any commercial or financial relationships that could be construed as a potential conflict of interest.

## Footnotes

1 http://imagej.nih.gov/ij/
