# Supplementary material for: Progranulin Adsorbs to Polypropylene Tubes and Disrupts Functional Assays: Implications for Research, Biomarker Studies, and Therapeutics
Source: Front Neurosci. 2020 Dec 14;14:602235. doi: 10.3389/fnins.2020.602235 (PMC7768044; doi:10.3389/fnins.2020.602235)
Supplement: Supplementary file 1 [file Table_1.DOCX]

**Manuscript Title**

Progranulin Adsorbs to Polypropylene Tubes and Disrupts Functional Assays: Implications for Research, Biomarker Studies, and Therapeutics

**Authors**

Sushmitha Gururaj^1^*, Paul J. Sampognaro^1^*, Andrea R. Argouarch^1^, Aimee W. Kao^1^

* These authors made equal contributions


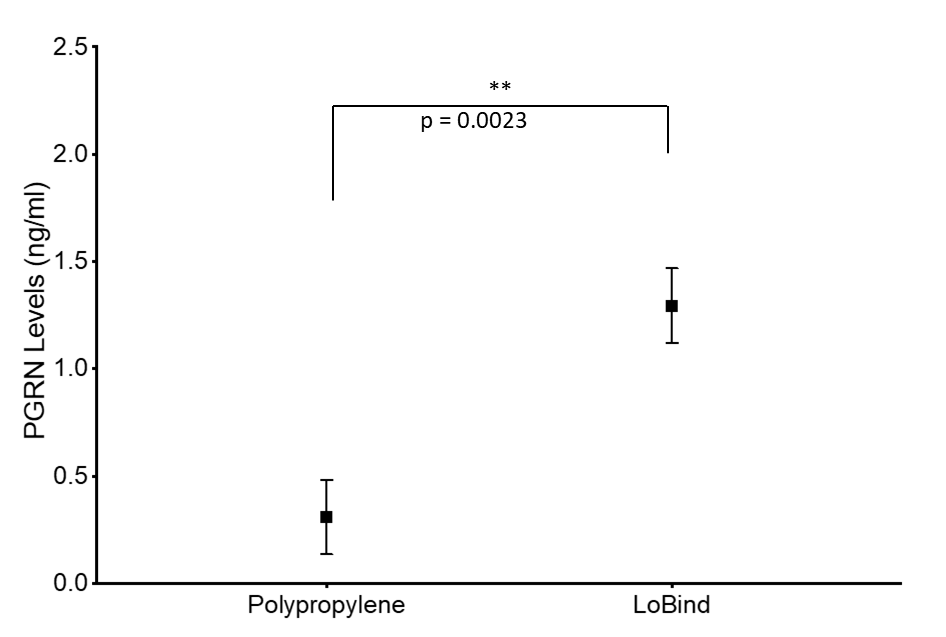


**Supplemental Figure 1. Progranulin Adsorbs to Polypropylene Tubes as Measured by ELISA.** 200ng/mL rhPGRN was incubated in either Polypropylene or LoBind tubes for 10 minutes. Solution was then taken from each tube and measured by ELISA using a 1:100 dilution. This method demonstrates similar results to our Western Blot quantitative measurements.


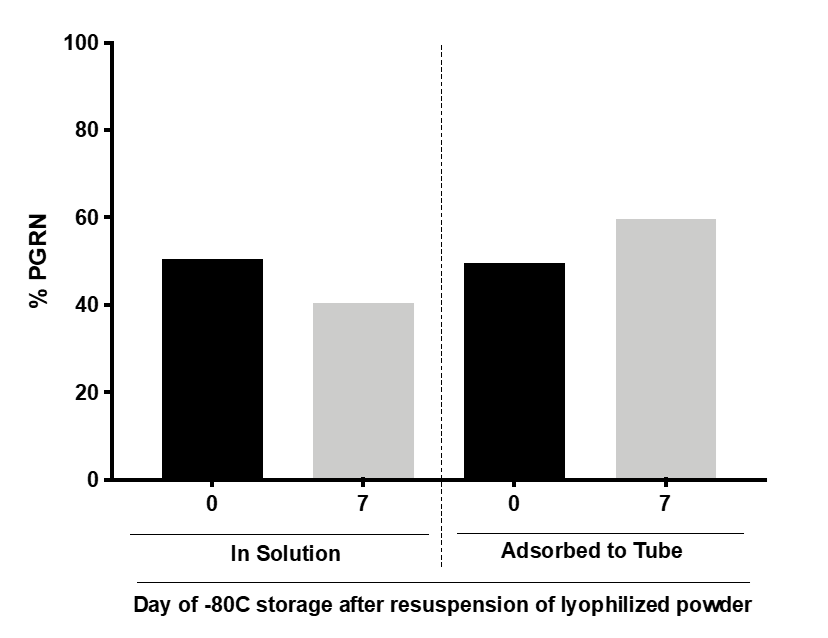


**Supplemental Figure 2. Progranulin Adsorption to Polypropylene Tubes after 1 week incubation at -80C for 1 week.** 100 nM PGRN was incubated for either 0 or 7 days in polypropylene tubes in a -80C freezer. At the end of the incubation periods, PGRN in solution as well as adsorbed to the tube was prepared for analysis. PGRN levels were measured by Western blot and membranes were immunoblotted with anti-PGRN antibody. All immunoblots are representative of three independent experiments.

**Supplemental Materials and Methods:**

**Progranulin enzyme-linked immunosorbent assays (ELISAs)**

Recombinant Progranulin (rhPGRN) samples (200ng/mL) were incubated in either polypropylene or Lobind tubes for 10 minutes and then measured using an ELISA kit (Adipogen Inc., Seoul, Korea) using a 1:100 dilution of the protein samples in 1× diluent following manufacturer's instructions. To increase accuracy all samples were analyzed twice in three independent experiments.
